# The Role of Intracellular Calcium for the Development and Treatment of Neuroblastoma

**DOI:** 10.3390/cancers7020811

**Published:** 2015-05-22

**Authors:** Noothan Jyothi Satheesh, Dietrich Büsselberg

**Affiliations:** Weill Cornell Medical College in Qatar, Qatar Foundation-Education City, POB 24144, Doha, Qatar; E-Mail: noothanjyothi@yahoo.co.in

**Keywords:** neuroblastoma, intracellular calcium([Ca^2+^]_i_), PI3K/AKT, ALK, FAK, NGF signalling, differentiation, apoptosis, proliferation, chemotherapeutic treatment, chemotherapy, drug resistance

## Abstract

Neuroblastoma is the second most common paediatric cancer. It develops from undifferentiated simpatico-adrenal lineage cells and is mostly sporadic; however, the aetiology behind the development of neuroblastoma is still not fully understood. Intracellular calcium ([Ca^2+^]_i_) is a secondary messenger which regulates numerous cellular processes and, therefore, its concentration is tightly regulated. This review focuses on the role of [Ca^2+^]_i_ in differentiation, apoptosis and proliferation in neuroblastoma. It describes the mechanisms by which [Ca^2+^]_i_ is regulated and how it modulates intracellular pathways. Furthermore, the importance of [Ca^2+^]_i_ for the function of anti-cancer drugs is illuminated in this review as [Ca^2+^]_i_ could be a target to improve the outcome of anti-cancer treatment in neuroblastoma. Overall, modulations of [Ca^2+^]_i_ could be a key target to induce apoptosis in cancer cells leading to a more efficient and effective treatment of neuroblastoma.

## 1. Introduction

Paediatric cancers—like neuroblastoma—transpire during the neonatal epoch of an infant [1]. Neuroblastoma are solid malignant tumours which are widely diagnosed during infancy [2,3,4] and are the second most common paediatric tumours representing about 10% of all paediatric cancers [5]. According to the childhood and adolescent cancer statistics published by the American Cancer Society (2014), neuroblastoma (7%) is the third most frequent cancer in childhood only preceded by acute lymphocytic leukaemia (26%) and cancers of the brain and CNS (21%) [6]. In the United Kingdom, neuroblastoma is slightly more frequent and counts for nearly 8% of the childhood malignancies [7]. The aetiology of neuroblastoma is not fully understood [7]. Its incidence rate is slightly higher in boys than girls with a ratio of 1.2 to 1 [6,7]. Its prevalence and severity varies in different ethnicities around the world with more cases being reported among whites than other ethnicities [6].

Neuroblastoma arises from undifferentiated simpatico-adrenal lineage cells which derive from neural crest cells [8]. The reason for the failure of these sympatico-adrenal lineages is still unknown. Neuroblast masses develop mostly at the abdomen (~65%) but are also found at chest (~20%), neck (~5%) or pelvis (~5%) [3]. Neuroblastoma is a highly heterogeneous tumour, which varies from relentless progression of the disease to spontaneous regression [5]. Like ganglio-neuroblastoma and ganglio-neuroma, it is categorized as a peripheral neuroblastic tumour [3,9]. Neuroblastoma occurs mostly sporadic, with familial neuroblastoma being rare (only 1%–2% of all neuroblastoma cases) [10].

The International Neuroblastoma Risk Group Staging System (INRGSS) has introduced a standardized classification of the disease in order to guide clinical trials at different regions across the world [11,12]. INRGSS divides the disease in four major stages L1, L2, M and MS (Table 1) [11,13], also referred as “very low”, “low”, “intermediate” and “risk” patients. Other classifications of neuroblastoma are based on the age, tumour differentiation histologic appearance and genetic factors including oncogene MYCN, aberrations in 11q and tumour cell ploidy [11,13].

**Table 1:** Four major stages in “the international neuroblastoma risk group staging system”.

Stage	Description
**L1**	Localized tumour without any detectable image-defined risk factors
**L2**	Localized tumour with one or more image defined risk factors
**M**	Metastatic disease
**MS**	Metastatic disease with metastases confined to skin, liver, and/or bone marrow (confined to children ≤ 18 month)

In physiology terms, cancers could be described as the end-result of either of eight vital cellular modifications which include the (1) escape of cell from apoptosis, (2) development of an autocrine system (for synthesizing growth factors resulting in a higher rate of proliferation), (3) development of an insensitivity to anti-growth signals, (4) uncontrolled replication potential, (5) incessant angiogenesis, (6) ability for tissue invasion and metastasis, (7) reprogramming of cellular energetics and (8) escape from immune cells[14,15]. Thus, the altered balance between apoptosis and proliferation could result in switching a normal cell to a malignant cell.

The secondary messenger calcium plays key role in regulating cellular processes and hence cellular calcium homeostasis is crucial. In cancer cells, the intracellular calcium concentration ([Ca^2+^]_i_) is in prominence as it modulates the apoptotic or proliferative pathways of the cell. Several studies have conferred the impact of anti-cancer drugs on [Ca^2+^]_i_ levels. In this review, the following chapters will highlight the essential role of [Ca^2+^]_i_ in the development and treatment of neuroblastoma by interacting and exerting its effect on cell survival signalling, differentiation, proliferation and apoptosis [16,17].

## 2. Role of [Ca^2+^]_i_ in Neuroblastoma

**Figure 1:**
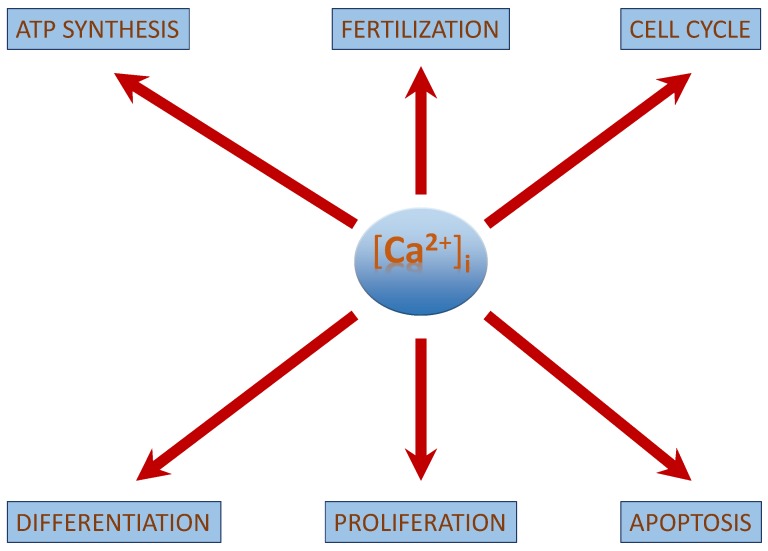
Role of [Ca^2+^]_i_:[Ca^2+^]_i_ regulates a range of cellular processes of which differentiation, proliferation and apoptosis is discussed for neuroblastoma. As the [Ca^2+^]_i_ signal defines the fate of the cell, it is critical to regulate its concentration.

[Ca^2+^]_i_ regulates multiple cellular processes such as fertilization, cell differentiation, proliferation, transcription factor activation, ATP-synthesis and apoptosis [18]. The concentration of [Ca^2+^]_i_ is vital to elicit a specific physiological function in normal functioning cells (Figure 1). This review discusses the mechanism by which [Ca^2+^]_i_ regulates particularly differentiation, proliferation and apoptosis in neuroblastoma and how its manipulation can be used to improve the efficiency of an anti-cancer therapy. It also focuses on the tumour suppressor role of cancer-sensing receptors (CaSR) in neuroblastoma. An overview on the role of [Ca^2+^]_i_ is discussed in Table 2. Increase in [Ca^2+^]_i_ is not restricted to neuroblastoma alone, as its found to have an apoptotic effect on breast cancer cellline MCF7 cells [19,20], making it a key molecule in regulation of cancers.

**Table 2:** An overview on the role of [Ca^2+^]_i_ in neuroblastoma.

[Ca^2+^]_i_ in Neuroblastoma
[Ca^2+^]_i_ interacts with the growth factor signaling cascade in neuroblastoma.
Three main kinases involved in cell survival signaling in neuroblastoma include PI3K/AKT, ALK and FAK.
[Ca^2+^]_i_ activated CAM kinases activates ERK1/2 exerts its role in neuroblastoma differentiation.
[Ca^2+^]_i_ regulated apoptosis in neuroblastoma involves the intrinsic pathway and the activation of CaSR.
Chemotherapeutic drug treatment shows an increase in [Ca^2+^]_i_ concentrations.

## 3. [Ca^2+^]_i_—Regulation and Signalling

**Figure 2:**
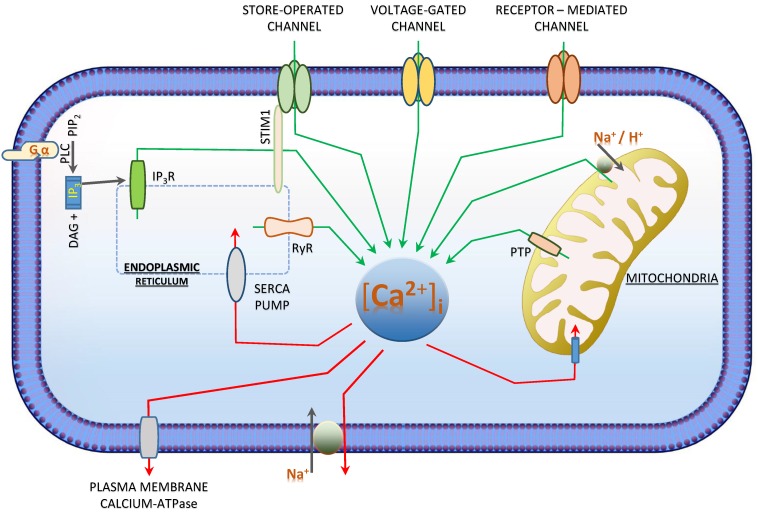
[Ca^2+^]_i_-Homeostasis by calcium influx and efflux: Intracellular calcium increases (green arrows) via the (1) voltage-gated channels, (2) receptor-mediated channels, (3) Na^2+^/Ca^2+^and Na^2+^/H^+^-Exchangers (4) store-operated channels which includes the IP_3_R and RYR (5) GPCR activated IP_3_R activation. [Ca^2+^]_i_ decreases (red arrows) via the (1) SERCA pump, (2) plasma membrane calcium ATPase pump (PMCA), (3) permeability transition pore (PTP) and (4) Na^2+^/Ca^2+^ Exchangers. In neuroblastoma, receptor activated for calcium influx are GPCRs (1) [24,25,26,27,28,29] and Sigma Receptors (2) [30]. Calcium release is more often from the stores (3) [24,25,27,28,29,31,32,33] or store-operated channel (4) [26].

Cells fate chiefly depends on the spatial, magnitude and temporal characteristic of [Ca^2+^]_i_ [17,21]. Resting cells have an [Ca^2+^]_i_ concentration between 10 and 100nM which could rise up to 100 times upon Ca^2+^-entry from the extracellular space or its release from the internal Ca^2+^-stores [18,22]. To maintain a low [Ca^2+^]_i_ concentration and to secure clearly defined [Ca^2+^]_i_ signals, calcium entry and extrusion mechanisms are tightly regulated [23]. As Ca^2+^ is high in the extracellular compartment as well as in the intracellular calcium stores, Ca^2+^-entry (from extracellular) or release (from the stores) will elevate the cytosolic [Ca^2+^]_i_-concentration [17,18]. [Ca^2+^]_i_ is reduced by active transport mechanisms which move the cytosolic calcium either back to the stores (Endoplasmic Reticulum (ER) and mitochondria) or the extracellular space (Figure 2). In cancer, [Ca^2+^]_i_ regulation mechanisms such as Na^+^/Ca^2+^-exchangers, calcium pumps and calcium channels are altered [17,21].

The influx mechanisms are regulated by voltage-, receptor- (binding of transmitters such as ATP, glutamate or acetylcholine to their corresponding receptors) and store-operated channels. As mentioned earlier, the internal stores of calcium includes ER or a similar organelle named sacroplasmic reticulum (SR) in muscle cells, of which the calcium release is mainly controlled by the inositol-1,4,5 triphosphate receptor (InsP3R) and the ryanodine receptor (RYR). Calcium is released from the ER by the binding of InsP3 and cyclic ADP ribose (cADPR) to the InsP3R and RYR, respectively. InsP3 is released in response to binding of hormones, growth factors and the neurotransmitteracetylcholine to their cell surface receptors by the cleavage of phosphatidyl inositol 4,5-bisphosphate, a phospholipid present in the cell membrane. In addition, Ca^2+^ is released from the ER by the nicotinic acid dinucleotide phosphate (NAADP) and sphingosine-1 phosphate (S1P) to the NAADP receptor and sphingolipid calcium release-mediating protein.

## 4. Signalling Pathways in Neuroblastoma and Their Dependence on [Ca^2+^]_i_

**Figure 3:**
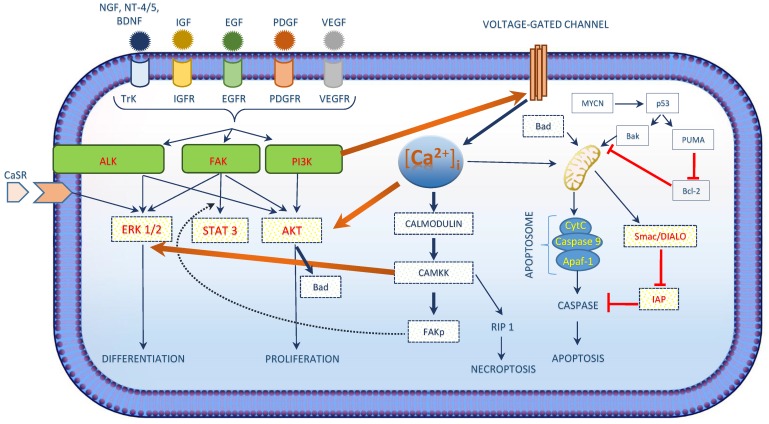
Intracellular calcium and cell survival: Intracellular calcium activates the intermediate proteins in the signalling pathways in neuroblastoma. Figure illustrates calcium regulating three kinases (AKT, ERK and FAK) that are involved in the cell survival signalling in neuroblastoma. The intracellular calcium and PI3K/AKT pathway influences one another forming a loop, while its impact on other two kinases is mainly via the activation of calmodulins and the CaM dependent protein kinase kinase.

Development of neuroblastoma tumourgenesis and malignancy arises due to unbalanced cell survival and apoptotic pathways. Nerve Growth Factor (NGF), Insulin-like Growth Factor (IGF), Epidermal Growth Factor (EGF), Platelet-Derived Growth Factor (PDGF) and Vascular Endothelial Growth Factor (VEGF) are major growth factor signalling pathways in Neuroblastoma (Figure 3). Cell survival signalling pathways are activated by these growth factors leading to the activation of downstream proteins by the intermediate kinases (PI3K/AKT, ALK and FAK) including the activation of transcription factors (MYCN, NF-KB and p53) (Figure 3) [34]. Of this, PI3K/AKT is of great importance as it is a major pathway involved in neuroblastoma development [35,36] and was described in primary neuroblastoma cells [37] and other cell lines (including SH-SY5Y, SK-N-SH, SK-N-BE, SK-N-EP and IMR32) [34,35]. PI3K/AKT promotes cell survival by activating survival associated proteins and by inhibiting the apoptotic pathway [38,39,40]. Several studies focused on the up-regulation of apoptosis in neuroblastoma cells by down regulation of the PI3K/AKT pathway and found that cells with MYCN amplification show a greater inhibition of the PI3K/AKT pathway which is considered to be a major factor for the prognosis of neuroblastoma [34,41,42]. Currently, AKT inhibitors including temsirolimus (a rapamycin analogue), perifosine (synthetic oral alkylphospholipid) are on clinical trials and its safety in children is being studied.

Anaplastic Lymphoma Kinase (ALK) is also associated with cell survival signalling of neuroblastoma. It belongs to the family of insulin receptors with the trans-membrane receptor tyrosine kinase [43] and exerts its role in cell growth and development, predominantly through the central nervous system [44]. In nearly 90% of neuroblastoma tumour samples, expression of ALK protein was observed [45] and was associated with ALK gene mutations [46]. ALK gene mutations are associated with both familial and sporadic neuroblastoma. Downstream signalling cascade of ALK signalling includes AKT, ERK1/2 and STAT3 [47].Calcium phosphorylates three kinases (AKT, ERK and FAK) that are involved in the cell survival signalling in neuroblastoma (Figure 3) [48,49].

### 4.1. [Ca^2+^]_i_ as a Key Factor which Determines the Fate of Neuroblastoma Cells

[Ca^2+^]_i_ also exert its effect on intermediate kinases that are involved in cell survival signalling. NGF signalling is a predominant signalling pathway in neuroblastoma. Trktyrosine kinase receptor family is associated with the NGF signalling and includes TrkA for NGF, TrkB for brain-derived neurotrophic factor (BDNF) and neurotrophin-4/5 (NT-4/5) and TrkC for neurotrophin-3 (NT-3) (Figure 3) [50]. Another receptor for NGF is p75^NTF^which exerts a lower affinity to NGF compared to TrkA. Expression of Trk in neuroblastoma varies with the type of neuroblastoma. High levels of TrkA and TrkC is expressed in biologically favourable neuroblastoma while high levels of TrkB and its ligand BDNF is expressed in biologically unfavourable aggressive neuroblastoma with MYCN amplification [51,52]. In PC12 cells, NGF ligand binding leads to the activation of ERK-MAP kinases for exerting its cellular functions. In addition, the NGF signal transduction is associated with an increase in [Ca^2+^]_i_ with a corresponding decrease in both internal stores and the extracellular spaces via the Trk receptors [53,54,55]. A study in monocytes and neurons revealed that PI3K/AKT pathway could phosphorylate Ca_V_β_2a_ which would end up being associated with the trafficking proteins and opening of voltage-gated calcium channels [56]. Thus, this increase in [Ca^2+^]_i_ could be due to the opening of the voltage-gated calcium channels across the plasma membrane by P13K [56] which is mainly activated in neuroblastoma.

ERK signalling cascade regulates cellular processes like development, differentiation and proliferation [57,58,59]. Both ERK signalling and elevated [Ca^2+^]_i_ are considered as key regulators for intracellular signalling initiated by extracellular ligands like growth factors and hormones as they determine the fate of the signalling pathway (Figure 3) [60]. In addition, extracellular ligand could lead to an increase of [Ca^2+^]_i_ [61,62] having an effect on the sub-cellular localization of ERK by interfering with its protein–protein interaction properties [60]. An elevated [Ca^2+^]_i_-concentration could either lead to activation or inhibition of ERK cascade signalling. Chuderland and co-workers showed that in rat cells, the translocation of phosphorylated ERKs from the cytosol to the nucleus is delayed by elevated [Ca^2+^]_i_, thereby affecting the localization of ERK [63]. However, molecular mechanisms to activate the ERK cascade by elevated [Ca^2+^]_i_ are more prevalent [64,65]. This is achieved by the activation of calmodulin dependent kinases 1 and 2 [66], PYK2 kinase [67], Ras-GRF [68] or by the inhibition of Ras-GAPs [69,70]. In support to the effect of [Ca^2+^]_i_on ERK signalling cascade, studies on PC12 cells also confirmed that activation of ERK by NGF signalling is regulated by both [Ca^2+^]_i_ and CaM[71]. Thus, the activation of ERK cascade activation by growth factor signalling is regulated by [Ca^2+^]_i_ and hence could have a role in neuroblastoma development as well. Studies using NG108 cells shows that an increase in [Ca^2+^]_i_ could help in cell survival by activating Protein Kinase B or AKT. Increased [Ca^2+^]_i_ activates Ca^2+^/CaM dependent protein kinase kinase (CaM-KK) which activates AKT thereby phosphorylating the serine residue 136 of BAD and hence promote cell survival [72].

FAK plays a key role in growth, survival, proliferation and migration of cells and is closely associated with neuroblastoma [34,73]. [Ca^2+^]_i_ phosphorylates FAK at its serine residues, as shown for Swiss 3T3 cells where [Ca^2+^]_i_ activates CaM-KII which leads to the activation of FAK by phosphorylating FAK at it Ser-843 residue (Figure 3).

### 4.2. [Ca^2+^]_i_ Induces Differentiation and Proliferation in Neuroblastoma

Neuroblastoma is associated with a block in cell differentiation, since the spontaneous regression of neuroblastoma is achieved partly through the neuronal differentiation of the cells. Treatment by inducing differentiation is considered as one of the most effective therapeutic strategies. Retinoic acid induces differentiation in neuroblastoma cell lines [2,74,75]. Currently, high-risk neuroblastoma patients are treated with retinoic acid and cells exhibit the inhibition of proliferation and the induction of differentiation [76]. The induction of differentiation in neuroblastoma cell lines is associated with an increase of [Ca^2+^]_i_.

Both neuroblastoma tumours and neuroblastoma cell lines consists of multipotent precursor cells which differentiate into discrete cell lineages of the neural crest [26,77]. Three main cellular phenotypes observed in neuroblastoma cell lines include neuroblastic N-type cells, substrate adherent S-type cells and intermediate I-type cells [78,79]. N-type cells consist of immature nerve cells and are precursors to the sympatho-adrenal cell lineage [26,79,80]. S-type cells are the non-neuronal lineage of the neural crest and are precursor cells of the Schwann, glial, and melanocytic cells of the neural crest [78,81]. With respect to the morphological characteristics and the biological markers, the I-type cells are considered to be intermediate to both N- and S-type cells [78,81]. I-type cells represent either a stem cell stage or an intermediate stage in between the trans-differentiation of N- and S-type cells [81,82]. N-type cells are more malignant in nature, while S-type cells are of a non-malignant nature [26,83,84].

An increase in [Ca^2+^]_i_ was observed in 9cRA-induced differentiation of SH-SY5Y and the differentiated N- and S-type cells from SH-SY5Y cells.SH-SY5Y cells shows a [Ca^2+^]_i_ concentration of 98 ± 4 nM and after treatment with 10 µM retinoic acid showed an increase in [Ca^2+^]_i_ [25]. These cells endogenously express muscarinic M3 and bardykinin B2 receptors and upon differentiation with 10 µM of retinoic acid followed by the stimulation of the receptors with methacholine and bradykinin resulted in greater elevation of [Ca^2+^]_i_ (Table 3) [85,86]. The [Ca^2+^]_i_ elevation measured 727 ± 30 nM and 535 ± 41 nM with the activation of 1mM methacholine and 10µM bradykinin. The authors concluded that the differentiation of these SH-SY5Y cells was associated with enhanced phosphoinositide signalling and that it was not receptor specific. Thus, this study confirmed that the increase of [Ca^2+^]_i_ was mediated by the release of Ca^2+^ from intracellular stores via Ins(1,4,5)P_3_ [25] (Figure 2).

Increase of [Ca^2+^]_i_ either from the extracellular space or from the internal stores upon treatment of neuroblastoma cell lines with several GPCR ligands like retinoic acid as in current neuroblastoma treatment, Sigma 2 ligands and other compounds like cisplatin, arsenic trioxide or tri-methyl-tin chloride (TMT) results either in differentiation or induction of apoptosis (Figure 2). This confers the role of [Ca^2+^]_i_ in inducing these pathways in the neuroblastoma cells and hence an insight into the possible role of [Ca^2+^]_i_ in the development and an efficient mechanism of action for the treatment of neuroblastoma.

### 4.3. [Ca^2+^]_i_ Induces Apoptosis in Neuroblastoma

**Figure 4:**
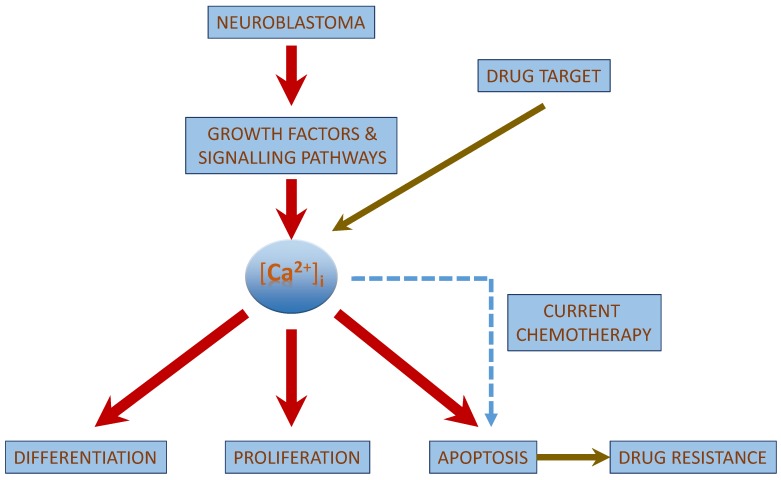
[Ca^2+^]_i_ as a possible drug target: [Ca^2+^]_i_ is involved in the signalling pathway of neuroblastoma pathophysiology and could be a possible drug target for the treatment of neuroblastoma.

Apoptosis is defined as programmed cell death (PCD) by which cells are eliminated in a biological system and is crucial for physiological processes like cell maintenance, development and tumour development and regression [87]. Apoptosis is induced either by extrinsic (death receptor) or intrinsic (mitochondrial) pathways, both leading to the activation of caspase 3, 6 and 7 which in turn cleave several other proteins and activate DNAses leading to cell death [50,88,89,90]. Diminution of cell apoptosis could be mediated by an altered balance of the pro- and anti-apoptotic proteins, decreased caspase activation and impaired extrinsic pathways. Studies revealed that under pathophysiological conditions, a greater quantity of mitochondrial Ca^2+^-uptake from the cytoplasm occurs thereby initiating cell apoptosis [91] (Figure 4). Mitochondrial uptake of Ca^2+^ from the ER also activates apoptosis with the opening of permeability transition pore (PTP) across the mitochondrial membrane by creating a negative mitochondrial membrane potential [92,93].

**Table 3:** Increase of [Ca^2+^]_i_ in human neuroblastoma cell lines: Color code in the table illustrates different receptors that initiates an increase of [Ca^2+^]_i_ in neuroblastoma cell lines of human origin. Two main categories of receptors mentioned include the G-protein coupled receptor and Sigma receptors (subtypes were not differentiated).

Sl.No	Cell Lines	Orgin	Treatment	Receptor	Concentration	Ca^2+^ Release	Basal [Ca^2+^]_i_	Increased [Ca^2+^]_i_	Reference
1	IMR-32	H	Orexin-A (GPCR)	Orexin Type 1 Receptor (GPCR)	3 nM	Store Release (IP_3_R)	50 nM	4 fold	[24]
2	SH-SY5Y	H	Retinoic Acid	Retinoid X receptor (Nuclear Receptors)	10 µM	Store Release	98 nM	No increase	[25]
3	SH-SY5Y	H	Retinoic Acid	Retinoid X receptor (Nuclear Receptors)	1 µM	Store Operated calcium Channel	10 nM	4 fold	[26]
4	SH-SY5Y	H	Oxotremorine-M	Muscarinic Receptor (GPCR)	10 µM	Store Release (IP_3_R)	50 nM	2 fold	[27]
5	SH-SY5Y	H	Methacholine	Muscarinic Receptor (GPCR)	1 mM	Store Release (IP_3_R)	98 nM	2 fold	[25]
6	SH-SY5Y	H	Carbachol	Muscarinic Receptor (GPCR)	1 mM	Store Release	-	3.5 fold	[28]
7	SK-N-SH	H	Carbachol	Muscarinic Receptor (GPCR)	100 µM	Store Release	59 nM	2 fold	[29]
8	SH-SY5Y	H	Bradykinin	Bradykinin Receptor (GPCR)	10 µM	Store Release (IP_3_R)	98 nM	1 fold	[25]
9	SH-SY5Y	H	Bradykinin	Bradykinin Receptor (GPCR)	10 µM	Store Release	-	2 fold	[28]
10	SH-SY5Y	H	Arsenic Trioxide	-	1 µM	Store Release (IP_3_R and RyR)	75 nM	2 fold	[31]
11	SH-SY5Y	H	Arsenic Trioxide	-	1 µM	Store Release (IP_3_R and RyR)	70 nM	3 fold	[32]
12	SH-SY5Y	H	Trimethyltin Chloride	-	0.1 µM	Store Release	-	2 fold	[33]
13	SH-SY5Y	H	Cisplatin	-	1 µM	Extracellular Space	75 nM	2 fold	[31]
14	SK-N-SH	H	CB-64D	Sigma 2 receptor	100 µM	Thapsigargin insensitive calcium store	-	4 fold	[30]
15	SK-N-SH	H	JL-II-147	Sigma 2 receptor	100 µM	Thapsigargin insensitive calcium store	-	2 fold	[30]
16	SK-N-SH	H	BD737	Sigma 2 receptor	100 µM	Thapsigargin insensitive calcium store	-	1 fold	[30]
17	SK-N-SH	H	LR172	Sigma 2 receptor	100 µM	Thapsigargin insensitive calcium store	-	1 fold	[30]
18	SK-N-SH	H	BD1008	Sigma 2 receptor	100 µM	Thapsigargin insensitive calcium store	-	1 fold	[30]
19	SK-N-SH	H	Haloperidol	Sigma 2 receptor	100 µM	Thapsigargin insensitive calcium store	-	1 fold	[30]
20	SK-N-SH	H	Ibogaine	Sigma 2 receptor	100 µM	Thapsigargin insensitive calcium store	-	1 fold	[30]

Mitochondrial Ca^2+^-uptake is channelled by mitochondrial inner membrane and the Ca^2+^-release is initiated by the mitochondrial outer membrane. Mitochondria Ca^2+^-Uniporters (MCU) are located in the mitochondrial inner membrane supporting the mitochondrial Ca^2+^-uptake across the membrane while creating a negative mitochondrial membrane potential. A mitochondrial membrane potential (Δψ_m_) of −180 mV developed across the membrane which facilitates MCU move Ca^2+^ across the membrane without interfering with the mitochondrial ATP hydrolysis or the transport of other ions [92,94]. Na^+^/Ca^2+^ and H^+^/Ca^2+^ exchangers support the transport of Ca^2+^ across the mitochondrial outer membrane. However, when Ca^2+^ is accumulated in the mitochondrial matrix it interacts with the prolyl isomerase cyclophilin D within the mitochondrial matrix resulting in the formation of PTP across the mitochondrial membrane [95]. This traverses the channel across the mitochondrial inner and outer membrane and allows Ca^2+^ (together with other ions and small molecules) to pass [92,96,97]. In addition, PTP opening is initiated by reactive oxygen species (ROS) and free fatty acids which are released with higher Ca^2+^-concentration in the matrix [98,99]. However, if the cytoplasm is filled with very high Ca^2+^-concentrations, PTPs remains open and permits the entry of solutes into the mitochondrial matrix which results in the expansion of the mitochondrial matrix. This results in the Mitochondrial Outer Membrane Permeabilization (MOMP) with the release of inter membrane space proteins like cytochrome c, Smac, DIABLO and caspase activation ensuing apoptotic cell death [100,101]. MOMP is sequestered with the activation of a cascade of both pro-apoptotic andanti-apoptotic members of the Bcl-2 family proteins [101].

Favourable neuroblastoma is often associated with a deregulated apoptotic pathway [50]. The sympathetic lineage neuroblast entails NGF, a neurotrophic factor for its cell survival and differentiation. Studies confirmed that the deletion or deficiency of these NGFs results in the activation of apoptotic cell death via intrinsic pathway in both normal cells and in tumour cells. Furthermore, when NGF is limited, apoptosis is triggered in favourable NB cells expressing TrkA and p75^NTF^ [50,102,103]. TrkB and their ligands are often related to MYCN amplification which is mainly associated with aggressive NB [50,104]. The genomic amplification of the transcription factor MYCN is a strong predictable clinical marker for the poor survival rate of the patients [105,106]. MYCN induces its role in differentiation, proliferation, cell growth, apoptosis, metabolism and protein synthesis [107]. Several studies reported that with the down regulation of MYCN expression in neuroblastoma cells induced cell apoptosis, morphological differentiation and cell growth arrest at the G1phase [108,109,110]. In the absence of NT-3, TrkC mediates apoptosis via the activation of caspase 9 [50,111], and since human aggressive NB is associated with higher expression of NT-3, caspase activated apoptosis is narrowed in these NBs [112]. These neurotrophins exert their survival role by Ras/MAPK and PI3K/AKT pathway [111,113].

Tumourgenesis is associated with the inactivation of p53, a classical tumour suppressor protein. In favourable NB, studies revealed the fact that apoptosis in NB is associated with p53.Nearly 25% of NB is associated with MYCN amplification. In MYCN amplified NB cell lines, cell apoptosis is mediated by p53 and is the direct transcriptional target of MYCN [114]. MYCN could also induce apoptosis in NB cells via activating the pro-apoptotic targets PUMA and Bax which are transactivated by p53 [114,115,116]. In addition, NB cells apoptosis is associated with MYCN amplification occurring via the activation of extrinsic apoptotic pathways [117,118].

In cultured cortical neurons, both BDNF and NT-3 lead to an elevation of [Ca^2+^]_i_ [119,120]. In addition, NGF induces an increase of Ca^2+^-uptake in both 3T3-Trk and 3T3-p75 cells in which the 3T3 cells are transfected with the high affinity NGF receptor p140^trk^and low affinity NGFR receptor p75^NGF^, respectively. 3T3-Trk also showed an increase of [Ca^2+^]_i_ irrespective of 3T3-p75 cells. Additionally, NGF helps in [Ca^2+^]_i_ signalling via voltage-gated channels in mouse nociceptive neurons. A suggestion by Bonnington and McNaughton is that out of the three pathways activated by NGF induced TrKA receptor activation, PI3K pathway leads to the sensitization of TRPV1. This sensitization increased Ca^2+^-influx via voltage-gated channels. Two other pathways activated by TrKA receptors are the PLC gamma and ShC induced Ras pathway [121]. Studies on basal forebrain neurons confirmed that upon culturing cells with NGF and BDNF for four to six days, a considerable increase in L- and N-type calcium channel currents were observed in NGF treated cells. Whereas, in BDNF treated cells, no change in calcium channel currents was observed [120]. Baldelli and co-workers describes that treatment of developing hippocampal neurons with NGF, NT-3 and BDNF for six to 20 days, voltage gated Ca^2+^-currents increased in a time and concentration dependent manner. BDNF exerted its Ca^2+^-influx via N, P/Q and R channels while NGF and NT-3 exerted its calcium influx via L-channels [122]. Others confirmed that calcium induces apoptosis via mitochondria [123,124].

Despite of the knowledge on MYCN amplification associated with neuroblastoma cell apoptosis, studies still confers the role of [Ca^2+^]_i_ in inducing intrinsic apoptotic pathway. In addition, studies have confirmed the role of [Ca^2+^]_i_ in inducing necroptosis in human neuroblastoma cells (Figure 3) via the activation of receptor interacting protein kinase 1 (RIP1) by the CAMKK [125].

### 4.4. Tumour Suppressor Functions of the Calcium-Sensing Receptor (CaSR) in Neuroblastoma

Calcium-sensing receptor (CaSR), a G-protein coupled receptor is a key mediator protein in maintaining the cellular responses and cell fate in response to external Ca^2+^-concentrations between 0.05–5 mM [126,127]. Change of extracellular Ca^2+^-concentration within this range transforms the proliferative cellular response to a stage of differentiation or quiescence [126]. In addition, the activation of CaSR is associated with an increased expression and secretion of parathyroid hormone-related peptide (PTHrP) which has a role in the development of hypercalcemia in cancer cells [126]. Loss or up-regulation of CaSR could result in the development of a neoplastic disease like cancer. However, CaSR activation exerts a different role in different types of cancers [9,128], as in prostate cancers, CaSR activation alleviates tumour cell progression and bone metastasis while in colon cancer; expression of CaSR is associated with differentiation in colon epithelium thereby acting as a tumour suppressor protein [129,130].

Recent studies with neuroblastoma brought more insights into calcium and calcium sensing receptors. The expression of CaSR and smaller levels of parathyroid hormone-related protein were observed in differentiated, favourable neuroblastic tumours [23,128]. CaSR exerts a tumour suppressor role in neuroblastoma [23]. Studies on human patient samples with relatively superior prognostic variables like low clinical stage and clinical risk and age of diagnosis below one year had confirmed CaSR expression on nearly 50% of both fully and partially differentiating neuroblastoma, along with the expression of PTHrP. CaSR expression was restricted mainly to the early phases of differentiation and only when the neuroblasts differentiation reaches a certain level, the PTHrP expression was observed [128].

In addition, Masdival and co-workers reported CaSR expression in developmental malignancy, confirming that both CaSR and PTHrP are expressed in differentiated favourable neuroblastoma and are unregulated upon inducing differentiation [23]. In undifferentiated unfavourable neuroblastoma, CaSR expression is silenced by several genetic and epigenetic mechanisms [23,128]. Also, *in vitro* experiments confirmed that when cells were exposed to Ca^2+^, cell vacuolization was observed along with an increase in number of dead cells. Thus, it was clear that CaSR was over expressed in neuroblastoma cells and when exposed to Ca^2+^ cells underwent apoptosis via ERK1/2signalling cascade [9].

Genome-wide association studies in relation to neuroblastoma were on focus and the susceptibility loci and alleles allied with the low and high risk neuroblastoma were revealed. The genetic variations on disease susceptibility included FLJ22536 (6p22) [23,131,132], BARD1(2q35) [23,132,133], LMO1(11p15) [23,132,134], HSD17B12 (11p11.2) [23,135], DUSP12(1q23.3) [23,135], LINC00340(6p22) [23,135], HACE1 and LIN28B(6q16) [23,136]. These studies confer that the genetic variations area plausible rationale for the development of benign and malignant neuroblastoma [133,135]. Genetic variants at the carboxy terminal of the CaSR were studied and three single nucleotide polymorphisms at rs1042636, rs1801725 and rs1801726 were observed and this tri-locus haplotype could be considered as an outcome predictor in neuroblastoma patients. The work also showed that the malignant nature of neuroblastoma is associated with the inactivation of CaSR gene in patients [23].

## 5. [Ca^2+^]_i_ Modulations with Chemotherapeutic Treatment of Neuroblastoma

Traditional treatment for neuroblastoma includes surgery, radiation therapy, chemotherapy, radiation therapy with stem cell rescue and the recent treatment therapies include targeted therapy and vaccines. Treatment protocols vary with the stage of disease (Table 4). Common chemotherapeutic drugs used for the neuroblastoma treatment includes Carboplatin, Cisplatin, Cyclophosphamide, Doxorubicin, Etoposide, Ifosfamide, Melphalan, Vincristine, Teniposide and Topotecan [137].

**Table 4:** Different stages in the development of neuroblastoma.

Stage of Neuroblastoma	Tumour Characteristics	Treatment Protocol
**Stage 1 (Low)**	Single site specific	Surgery
**Stage 2A (Low)**	Single site specific and could not be removed completely by surgery.	Surgery and Chemotherapy
**Stage 2B (Low)**	Single site specific and could be removed completely by surgery. Cancer development could be present at lymph nodes around the tumour.	Surgery
**Stage 3 (Intermediate Risk)**	Cancer could be present in one or both sides of the body and lymph nodes.	Chemotherapy
**Stage 4 (High Risk)**	Cancer spread to distant body parts (bone, liver, skin, bone marrow and other organs) and distant lymph nodes.	Surgery, Chemotherapy, Radiotherapy, Immunotherapy and Retinoid Therapy
**Stage 4S (High Risk)**	Child is younger than 12 months with cancer spread on one side of the body. Lymph nodes on the same side of the body also affected.	Surgery, Chemotherapy and Radiotherapy
**Relapsed/Recurrent**	-	Chemotherapy, Immunotherapy, Retinoid Therapy, Tyrosine kinase and Aurora kinase inhibitors and targeted delivery of radionuclides.

Chemotherapeutic drugs used for the treatment of cancer modulate [Ca^2+^]_i_ with the induction of necrosis or apoptosis. Studies report that arsenic trioxide (As_2_O_3_) and cisplatin (CDDP) [31,32] and trimethyltin chloride (TMT) [33] induce apoptosis in neuroblastoma cells by interfering with [Ca^2+^]_i_-homeostasis. Cisplatin is considered as the most effective chemotherapeutic drug for the treatment of neuroblastoma. Cisplatin exerts its anti-cancer therapeutic role by cytotoxicity (interacting with the purine bases in DNA at the nucleophilic N7-sites forming a interstrand and intrastrand of DNA-DNA and DNA-protein crosslinks) [138] and apoptosis (increase in activity of caspase-8 and caspase 9) [139]. Ciplatin also interacts with calcium signaling, p53 and reactive oxygen species [138,139] and apoptosis by an increase in activity of caspase-8 and caspase 9 and up regulation ofp53 expression [22]. Followed by the application, [Ca^2+^]_i_ was elevated by Ca^2+^ entry from the extracellular space [22,139]. The studies concluded that the rise of [Ca^2+^]_i_ was CDDP-concentration dependent, leading to apoptosis by the activation of calpain mediated by ionsitol tri-phosphate (IP_3_) [22,139]. It was suggested that by increasing [Ca^2+^]_i_ through a different activation of IP_3_, the efficacy of cisplatin could be increased resulting in a higher rate of apoptosis. Cisplatin increased [Ca^2+^]_i_ by Ca^2+^-entry and As_2_O_3_ by Ca^2+^-release from the internal stores [32,140]. Cisplatin induced increase in [Ca^2+^]_i_ is allied with a cellular apoptosis with the activation of calpain and not caspase-8, confirming that cisplatin induced calcium influx via the IP_3_ receptors lead to cellular apoptosis with the activation of calpain [22].

## 6. Drug Resistance in Neuroblastoma

Despite of the above mentioned advanced treatment in neuroblastoma, treatment protocols are often ineffective in patients with aggressive neuroblastoma due to the development of drug resistance to chemotherapeutic drugs [141,142] (Figure 4). Drug resistance leads to a greater rate of disease relapse than normally expected, and in neuroblastoma, resistance is often related to the expression of a multi-drug resistant-associated protein (MRP1) [141,143] or due to a mutations or loss of function of p53 [144]. However, apart from the drug resistance, chemotherapeutic treatment could also lead to an increase in malignancy and metastasis of the disease, thereby affecting its biological properties [142].

Studies on resistant neuroblastoma cell lines UKF-NB-2rVCR20 and UKF-NB-2rDOX100 showed a greater resistance to vincristine (VCR) and doxorubicin (DOX), respectively, whereas these cell lines still responded to cisplatin. In addition, with respect to the normal cell lines, these cell lines showed a variation in the expression levels of P-glycoprotein, GD2 and neural cell adhesion molecule (NCAM), an adhesion receptor resulting in a greater rate of tumourgenesis in *in vivo* studies [145]. Drug resistance also leads to an increase in adhesion of the resistant neuroblastoma cell lines to the endothelium [145,146]. As_2_O_3_ is more effective in normoxia and hypoxia than the common drug etoposide in multi-drug resistance cell lines. Thus, As_2_O_3_ could be considered as a more effectual drug in the treatment of patients with aggressive neuroblastoma where a greater level of hypoxia is usually observed. Therefore, it is vital to identify the molecular mechanism behind the development of drug resistance as it will help to design new chemotherapeutic drug(s) with better efficacy and also will help to predict the possible outcomes of a new drug when introduced [147].

## 7. Future Studies with [Ca^2+^]_i_ in Neuroblastoma

The role of [Ca^2+^]**_i_** in neuroblastoma development, proliferation and apoptosis can be better understood when the complexity behind the Ca^2+^-concentration regulating the cellular process is considered. In neuroblastoma, [Ca^2+^]_i_ is a key mediator and might not be restricted to growth factor signalling and the kinase activation. Apart from these, studies relating to [Ca^2+^]**_i_** on MYCN expression is questionable as neuroblastoma is often associated with MYCN over expression. Since [Ca^2+^]**_i_** rises with chemotherapeutic treatment, elevating [Ca^2+^]_i_ agonist could improve the current treatment protocol. However, the studies should be extended to other biological and environmental factors like Vitamin D deficiency and calcium absorption as the disease prevalence is higher in regions with less sunlight. In addition, further insights into calcium deficiency in pregnancy and the scope of [Ca^2+^]_i_ in treatment of other types of cancers are to be discussed.

## 8. Conclusions

[Ca^2+^]_i_ regulates different cellular functions and has a key role in the development and prevalence of neuroblastoma. In neuroblastoma, these [Ca^2+^]_i_ form a network with the intermediate kinase of major signalling pathways, either directly or indirectly, and its concentration determines the fate of the cells. Hence, studies could be more focused on [Ca^2+^]_i_, which could lead to the development of new Ca^2+^ based drugs or could also help in improving the efficacy of chemotherapeutic drugs that are currently used in treatment of neuroblastoma.

## Abbreviations

INRGSS: International Neuroblastoma Risk Group Staging System
Ca^2+^: Calcium Ions
[Ca^2+^]_i_: Intracellular Calcium
CaSR: Cancer-Sensing Receptors
ATP: Adenosine Triphosphate
ER: Endoplasmic Reticulum
IP_3_: Ionsitol Triphosphate
InsP3R: Inositol-1,4,5 Triphosphate Receptor
RyR: Ryanodine Receptor
NAADP: Nicotinic Acid Dinucleotide Phosphate
S1P: Sphingosine-1 Phosphate
cADPR: Cyclic ADP Ribose
NGF: Nerve Growth Factor
IGF: Insulin-like Growth Factor
EGF: Epidermal Growth Factor
PDGF: Platelet-derived Growth Factor
VEGF: Vascular Endothelial Growth Factor
ALK: Anaplastic Lymphoma Kinase
BDNF: Brain-Derived Neurotrophic Factor
NT-4/5: Neurotrophin-4/5
MCU: Mitochondria Calcium Uniporter
Δψ_m_: Mitochondrial Membrane Potential
MOMP: Mitochondrial Outer Membrane Permeabilization
As_2_O_3_: Arsenic Trioxide
TMT: Trimethyltin Chloride
MRP1: Multi-Drug Resistant-Associated Protein
VCR: Vincristine
DOX: Doxorubicin
NCAM: Neural Cell Adhesion Molecule
